# Data on assessing fluoride risk in bottled waters in Iran

**DOI:** 10.1016/j.dib.2018.08.160

**Published:** 2018-08-31

**Authors:** Mahmood Alimohammadi, Ramin Nabizadeh, Kamyar Yaghmaeian, Amir Hossein Mahvi, Peyman Foroohar, Saeedeh Hemmati, Zoha Heidarinejad

**Affiliations:** a Center for Water Quality Research (CWQR), Institute for Environmental Research (IER), Tehran University of medical sciences, Tehran, Iran; b Department of Environmental Health Engineering, School of public health, Tehran University of medical sciences, Tehran, Iran; c Health Equity Research Center (HERC) Tehran University of Medical Sciences, Tehran, Iran; d Iranian Bottled Water Association, Tehran, Iran; e Food Health Research Center, Hormozgan University of Medical Sciences, Bandar Abbas, Iran

**Keywords:** Bottled water, Risk assessment, Fluoride, Iran

## Abstract

The general goal of this data was to determine the concentration of fluoride and assess its risk in waters bottled in Iran. Seventy-one samples of different brands of bottled water were collected. Then, the fluoride concentration was measured through standard method for water and wastewater experiments. The non-carcinogenicity risk of fluoride for different groups of infants, children, teenagers, and adults was calculated by proper formula. The data presented here indicated that the mean concentration of fluoride in bottled waters was 0.272 mg/L, which is lower than the minimum world health organization (WHO) guideline. Further, the mean hazard quotient (HQ) values for fluoride across the groups of infants, children, teenagers, and adults with respective values of 0.0363, 0.2568, 0.1813, and 0.1452 were observed in 0, 1, 1, and 0 cases of HQ>1. Generally, in most of the tested brands, HQ value was less than 1, and this value was above 1 in only one brand of bottled water.

**Specifications Table**

**Table:** 

Subject area	Bottled water quality and risk assessment
More specific subject area	Bottled water fluoride
Type of data	Tables
How data was acquired	Bottled water brands tested were obtained from the Iranian Bottled water association. The fluoride concentration of the samples was measured using an Ion Chromatography No. 4110 in accordance with the method described in “Standard methods: For the examination water and wastewater, 22nd edn” [1], [2], [3], [4], [5], [6], [7], [8].
Data format	Raw, Analyzed
Experimental factors	71 different brands of high-consumption bottled water in Iran were randomly selected
Experimental features	Determine the concentration level of fluoride
Data source location	Iran
Data accessibility	The data are available with this article

**Value of the data**

• The data analysis indicated that HQ value was less than one in many of the studied brands. Therefore, the possibility of incidence of non-carcinogenicity effects resulting from fluoride consumption in bottled waters was very low, and presence of fluoride in the bottled waters does not pose any risk to the consumers.
• This data can be useful to consumers of bottled water in terms of quality of bottled water help to consumers in areas with low concentrations of fluoride in drinking water, food and beverages with high levels of fluoride use.
• Since water deficit exists in some regions of Iran, thus people use bottled waters. As the mean concentration of fluoride is lower than the standard value in many of the measured water samples, thus it is suggested that fluoride-containing toothpastes be used in these regions.
• This data can be used by researchers and managers of bottled waters production industries to evaluate the effects of fluoride in bottled waters and improve the quality of bottled waters.

## 1 Data

The parameters used in calculation of fluoride risk assessment in the consumed bottled waters are provided in Table 1. Fluoride concentration and fluoride estimated daily intake (EDI) and the values of hazard quotient (HQ) for the four population groups consuming bottled waters are shown in Table 2.

**Table 1:** Parameters used in the present study data for health exposure assessment in drinking water [35].

Parameter	Risk exposure factors	Values for groups				Unit
		Infants	Children	Teenagers	Adults	
Fluoride	C_f_	–	–	–	–	mg/L
	C_d_	0.08	0.85	2	2.5	L/day
	B_w_	10	15	50	78	Kg
	RfD	0.06	0.06	0.06	0.06	mg/kg day

**Table 2:** Fluoride concentration (mg/L) and fluoride estimated daily intake (EDI) and hazard quotient (HQ) for the four populations of bottled water consumers.

Nos	Fluoride	EDI						HQ					
	concentration	Infants	Children	Teenagers		Adults		Infants	Children		Teenagers	Adults	
101	0.4	0.0032	0.0227		0.016		0.0128	0.0533		0.3778	0.2667		0.2137
102	0.198	0.0016	0.0112		0.0079		0.0063	0.0264		0.187	0.132		0.1058
103	1.553	0.0124	0.088		0.0621		0.0498	0.2071		14.667	10.353		0.8296
104	0.23	0.0018	0.013		0.0092		0.0074	0.0307		0.2172	0.1533		0.1229
105	0.6	0.0048	0.034		0.024		0.0192	0.08		0.5667	0.4		0.3205
106	0.041	0.0003	0.0023		0.0016		0.0013	0.0055		0.0387	0.0273		0.0219
107	0.552	0.0044	0.0313		0.0221		0.0177	0.0736		0.5213	0.368		0.2949
108	0.3705	0.003	0.021		0.0148		0.0119	0.0494		0.3499	0.247		0.1979
109	0.79	0.0063	0.0448		0.0316		0.0253	0.1053		0.7461	0.5267		0.422
110	0.002	0	0.0001		0.0001		0.0001	0.0003		0.0019	0.0013		0.0011
111	0.1185	0.0009	0.0067		0.0047		0.0038	0.0158		0.1119	0.079		0.0633
112	0.269	0.0022	0.0152		0.0108		0.0086	0.0359		0.2541	0.1793		0.1437
113	0.431	0.0034	0.0244		0.0172		0.0138	0.0575		0.4071	0.2873		0.2302
114	0.24	0.0019	0.0136		0.0096		0.0077	0.032		0.2267	0.16		0.1282
115	0.16	0.0013	0.0091		0.0064		0.0051	0.0213		0.1511	0.1067		0.0855
116	0.0865	0.0007	0.0049		0.0035		0.0028	0.0115		0.0817	0.0577		0.0462
117	0.588	0.0047	0.0333		0.0235		0.0188	0.0784		0.5553	0.392		0.3141
118	0.2365	0.0019	0.0134		0.0095		0.0076	0.0315		0.2234	0.1577		0.1263
119	0.365	0.0029	0.0207		0.0146		0.0117	0.0487		0.3447	0.2433		0.195
120	0.2335	0.0019	0.0132		0.0093		0.0075	0.0311		0.2205	0.1557		0.1247
121	0.1	0.0008	0.0057		0.004		0.0032	0.0133		0.0944	0.0667		0.0534
122	0.105	0.0008	0.006		0.0042		0.0034	0.014		0.0992	0.07		0.0561
123	0.3265	0.0026	0.0185		0.0131		0.0105	0.0435		0.3084	0.2177		0.1744
124	0.2135	0.0017	0.0121		0.0085		0.0068	0.0285		0.2016	0.1423		0.114
125	0.0825	0.0007	0.0047		0.0033		0.0026	0.011		0.0779	0.055		0.0441
126	0.272	0.0022	0.0154		0.0109		0.0087	0.0363		0.2569	0.1813		0.1453
127	0.074	0.0006	0.0042		0.003		0.0024	0.0099		0.0699	0.0493		0.0395
128	0.1355	0.0011	0.0077		0.0054		0.0043	0.0181		0.128	0.0903		0.0724
129	0.58	0.0046	0.0329		0.0232		0.0186	0.0773		0.5478	0.3867		0.3098
130	0.142	0.0011	0.008		0.0057		0.0046	0.0189		0.1341	0.0947		0.0759
131	0.24	0.0019	0.0136		0.0096		0.0077	0.032		0.2267	0.16		0.1282
132	0.3965	0.0032	0.0225		0.0159		0.0127	0.0529		0.3745	0.2643		0.2118
133	0.486	0.0039	0.0275		0.0194		0.0156	0.0648		0.459	0.324		0.2596
134	0.5565	0.0045	0.0315		0.0223		0.0178	0.0742		0.5256	0.371		0.2973
135	0.301	0.0024	0.0171		0.012		0.0096	0.0401		0.2843	0.2007		0.1608
136	0.191	0.0015	0.0108		0.0076		0.0061	0.0255		0.1804	0.1273		0.102
137	0.2565	0.0021	0.0145		0.0103		0.0082	0.0342		0.2423	0.171		0.137
138	0.305	0.0024	0.0173		0.0122		0.0098	0.0407		0.2881	0.2033		0.1629
139	0.281	0.0022	0.0159		0.0112		0.009	0.0375		0.2654	0.1873		0.1501
140	0.4155	0.0033	0.0235		0.0166		0.0133	0.0554		0.3924	0.277		0.222
141	0.526	0.0042	0.0298		0.021		0.0169	0.0701		0.4968	0.3507		0.281
142	0.272	0.0022	0.0154		0.0109		0.0087	0.0363		0.2569	0.1813		0.1453
143	0.1555	0.0012	0.0088		0.0062		0.005	0.0207		0.1469	0.1037		0.0831
144	0.095	0.0008	0.0054		0.0038		0.003	0.0127		0.0897	0.0633		0.0507
145	0.033	0.0003	0.0019		0.0013		0.0011	0.0044		0.0312	0.022		0.0176
146	0.408	0.0033	0.0231		0.0163		0.0131	0.0544		0.3853	0.272		0.2179
147	0.15	0.0012	0.0085		0.006		0.0048	0.02		0.1417	0.1		0.0801
148	0.154	0.0012	0.0087		0.0062		0.0049	0.0205		0.1454	0.1027		0.0823
149	0.272	0.0022	0.0154		0.0109		0.0087	0.0363		0.2569	0.1813		0.1453
150	0.389	0.0031	0.022		0.0156		0.0125	0.0519		0.3674	0.2593		0.2078
151	0.207	0.0017	0.0117		0.0083		0.0066	0.0276		0.1955	0.138		0.1106
152	0.272	0.0022	0.0154		0.0109		0.0087	0.0363		0.2569	0.1813		0.1453
153	0.119	0.001	0.0067		0.0048		0.0038	0.0159		0.1124	0.0793		0.0636
154	0.973	0.0078	0.0551		0.0389		0.0312	0.1297		0.9189	0.6487		0.5198
155	0.108	0.0009	0.0061		0.0043		0.0035	0.0144		0.102	0.072		0.0577
156	0.027	0.0002	0.0015		0.0011		0.0009	0.0036		0.0255	0.018		0.0144
157	0.237	0.0019	0.0134		0.0095		0.0076	0.0316		0.2238	0.158		0.1266
158	0.307	0.0025	0.0174		0.0123		0.0098	0.0409		0.2899	0.2047		0.164
159	0.1	0.0008	0.0057		0.004		0.0032	0.0133		0.0944	0.0667		0.0534
160	0.082	0.0007	0.0046		0.0033		0.0026	0.0109		0.0774	0.0547		0.0438
161	0.473	0.0038	0.0268		0.0189		0.0152	0.0631		0.4467	0.3153		0.2527
162	0.2	0.0016	0.0113		0.008		0.0064	0.0267		0.1889	0.1333		0.1068
163	0.084	0.0007	0.0048		0.0034		0.0027	0.0112		0.0793	0.056		0.0449
164	0.276	0.0022	0.0156		0.011		0.0088	0.0368		0.2607	0.184		0.1474
165	0.141	0.0011	0.008		0.0056		0.0045	0.0188		0.1332	0.094		0.0753
166	0.046	0.0004	0.0026		0.0018		0.0015	0.0061		0.0434	0.0307		0.0246
167	0.082	0.0007	0.0046		0.0033		0.0026	0.0109		0.0774	0.0547		0.0438
168	0.05	0.0004	0.0028		0.002		0.0016	0.0067		0.0472	0.0333		0.0267
169	0.069	0.0006	0.0039		0.0028		0.0022	0.0092		0.0652	0.046		0.0369
170	0.043	0.0003	0.0024		0.0017		0.0014	0.0057		0.0406	0.0287		0.023
171	0.03	0.0002	0.0017		0.0012		0.001	0.004		0.0283	0.02		0.016
Min	0.002	0	0.0001		0.0001		0.0001	0.0003		0.0019	0.0013		0.0011
Max	1.553	0.0124	0.088		0.0621		0.0498	0.2071		14.667	10.353		0.8296
Mean	0.272	0.0022	0.0154		0.0109		0.0087	0.0363		0.2568	0.1813		0.1452
SD	0.245	0.002	0.0139		0.0098		0.0078	0.0326		0.2312	0.1632		0.1307

## 2 Experimental design, materials and methods

### 2.1 Measuring the fluoride concentration in bottled waters

Seventy one samples of different brands of bottled waters were chosen randomly, and then the fluoride concentration of water samples was determined by Ion Chromatography No. 4110 based on the method mentioned in the book of standard methods for water and wastewater experiments [8], [9], [10], [11], [12], [13], [14], [15], [16], [17], [18], [19], [20], [21], [22], [23], [24], [25], [26], [27], [28], [29].

### 2.2 Assessing fluoride risk

In this data, the non-carcinogenicity risk of fluoride resulting from consuming bottled waters was calculated across different age groups. First, the parameters of classification of age groups, body weight, and amount of water consumption were chosen based on the study by Yousefi et al., such that the age groups were categorized into the groups of infants (younger than two years old), children (2–6 years old), teenagers (6–16 years old) and adults (above 16 years of age), and the mean water consumption across the age groups was selected as 0.08, 0.85, 2, and 2.5 L/day, respectively [25]. The body weight across the different age groups of newborns, children, teenagers, and adults was chosen as 10, 15, 50, and 75 kg, respectively.

Then, estimation of data consumption of fluoride across the different brands of bottled waters was performed by Eq. (1) [25], [30], [31], [32], [33], [34]:

(1) $$\mathrm{EDI}=\frac{{C_{f}\times C}_{d}}{B_{w}}$$

EDI: Estimated daily intake of fluoride (mg/kg d),

C_f_: The fluoride concentration in the bottled waters (mg/kg),

C_d_: The mean daily consumption of drinking water (L/d),

B_w_: Body weight (kg).

Furthermore, the hazard quotient (HQ) was calculated by Eq. (2) to predict the non-carcinogenicity risk of exposure to fluoride:

(2) $$\mathrm{HQ}=\frac{\mathrm{EDI}}{\mathrm{RFD}}$$

In this equation, HQ represents the hazard quotient (to show the non-carcinogenicity effects), CDI is the chronic daily intake (mg/kg d), and RFD is the reference dose (mg/kg.d). The reference dose for fluoride is 0.06 mg/kg d.

HQ value larger than 1 suggests probability of incidence of non-carcinogenicity effects in the exposed population. On the other hand, HQ<1 shows absence of probability of incidence of non-carcinogenicity effects in the exposed population.
